# The radiological characteristics, tertiary lymphoid structures, and survival status associated with EGFR mutation in patients with subsolid nodules like stage I-II LUAD.

**DOI:** 10.1186/s12885-024-12136-6

**Published:** 2024-03-25

**Authors:** Mei Xie, Jie Gao, Xidong Ma, Jialin Song, Chongchong Wu, Yangyu Zhou, Tianjiao Jiang, Yiran Liang, Chen Yang, Xinyu Bao, Xin Zhang, Jie Yao, Ying Jing, Jianlin Wu, Jianxin Wang, Xinying Xue

**Affiliations:** 1 Department of Respiratory and Critical Care, Chinese PLA General Hospital, the First Medical Centre, 100835 Beijing, People’s Republic of China; 2 Department of Pathology, Chinese PLA General Hospital, the First Medical Centre, 100835 Beijing, People’s Republic of China; 3 Department of Respiratory and Critical Care, Beijing Shijitan Hospital, Capital Medical University, 100038 Beijing, People’s Republic of China; 4 Department of Respiratory and Critical Care, Weifang Medical College, 261053 Weifang, People’s Republic of China; 5 Department of Radiology, Chinese PLA General Hospital, the First Medical Centre, 100835 Beijing, People’s Republic of China; 6 Department of Radiology, Affiliated Hospital of Qingdao University, 266500 Qingdao, People’s Republic of China; 7 Department of Laboratory Medicine, Chinese PLA General Hospital, the First Medical Centre, 100835 Beijing, People’s Republic of China; 8 Center for Intelligent Medicine, Greater Bay Area Institute of Precision Medicine (Guangzhou), School of Life Sciences, Fudan University, 510000 Guangzhou, People’s Republic of China; 9 Department of Radiology, Affiliated Zhongshan Hospital of Dalian University, 116001 Dalian, People’s Republic of China

**Keywords:** LUAD, Subsolid nodule, Epidermal growth factor receptor, Tertiary lymphoid structures, Computed tomography

## Abstract

**Background:**

Epidermal growth factor receptor tyrosine kinase inhibitors (EGFR-TKIs) recommended for the patients with subsolid nodule in early lung cancer stage is not routinely. The clinical value and impact in patients with EGFR mutation on survival outcomes is further needed to be elucidated to decide whether the application of EGFR-TKIs was appropriate in early lung adenocarcinoma (LUAD) stage appearing as subsolid nodules.

**Materials and methods:**

The inclusion of patients exhibiting clinical staging of IA-IIB subsolid nodules. Clinical information, computed tomography (CT) features before surgical resection and pathological characteristics including tertiary lymphoid structures of the tumors were recorded for further exploration of correlation with EGFR mutation and prognosis.

**Results:**

Finally, 325 patients were enrolled into this study, with an average age of 56.8 ± 9.8 years. There are 173 patients (53.2%) harboring EGFR mutation. Logistic regression model analysis showed that female (OR = 1.944, *p* = 0.015), mix ground glass nodule (OR = 2.071, *p* = 0.003, bubble-like lucency (OR = 1.991, *p* = 0.003) were significant risk factors of EGFR mutations. Additionally, EGFR mutations were negatively correlated with TLS presence and density. Prognosis analysis showed that the presence of TLS was associated with better recurrence-free survival (RFS)(*p* = 0.03) while EGFR mutations were associated with worse RFS(*p* = 0.01). The RFS in patients with TLS was considerably excel those without TLS within EGFR wild type group(*p* = 0.018). Multivariate analyses confirmed that EGFR mutation was an independent prognostic predictor for RFS (HR = 3.205, *p* = 0.037).

**Conclusions:**

In early-phase LUADs, subsolid nodules with EGFR mutation had specific clinical and radiological signatures. EGFR mutation was associated with worse survival outcomes and negatively correlated with TLS, which might weaken the positive impact of TLS on prognosis. Highly attention should be paid to the use of EGFR-TKI for further treatment as agents in early LUAD patients who carrying EGFR mutation.

## Introduction

Lung cancer is the major cause of high morbidity and mortality in the cancer spectrum. Among all cancer cases, non-small cell lung cancer (NSCLC) accounts for over 85%. Lung adenocarcinoma (LUAD) is the most common histologic subtype of NSCLC [1–3]. Epidermal growth factor receptor (EGFR) was a high mutation rate genes and considered to have important roles in progression of LUAD appearing as subsolid nodules [4, 5]. In the last decade, the development of molecular-targeted therapy has led to impressive tumor remission in advanced NSCLC patients and improved the patient prognosis with lower toxicity risks than platinum-based chemotherapy [6–8]. Of note, NSCLC patients carrying EGFR mutations had astonishing response rates to EGFR tyrosine kinase inhibitors (EGFR-TKIs), such as osimertinib [9].

With the increasing amount of available targeted therapies, the therapeutic strategies for early-stage LUAD are also evolving [10–12]. Previously, early-stage lung cancer patients are treated only through surgical resection alone. Recently, the phase III ADAURA clinical trial has certificated a dramatically better clinical benefits in disease-free survival (DFS) of patients in stage IB-IIIA LUAD harboring EGFR mutations treated by osimertinib after completely resection of tumor compared with placebo group [13, 14]. Osimertinib hence has been successfully recommended by National Comprehensive Cancer Network (NCCN) guidelines for the adjuvant targeted setting for EGFR-mutation positive NSCLC [15]. EGFR mutation status analysis becomes one of the important factors in therapeutic strategies for lung cancer and is recommended for tumor resection, even the earliest stage lung cancer [16].

However, the EGFR-TKIs used for early-stage lung cancer is not routinely. The influence and clinical value of EGFR mutation on survival outcomes is still need to be elucidated to decide whether the EGFR-TKIs was appropriate or not for all early-stage LUAD appearing as subsolid nodules. In additional, it is also essential to demonstrated the correlation between EGFR mutation and presence of tertiary lymphoid structures in immune microenvironment, which is reported to correlated with favorable prognosis in resectable NSCLC. Thus, this retrospective report was performed to probe the influence of EGFR mutation on survival outcomes, tertiary lymphoid structures and radiological features in stage I-II LUAD patients appearing as subsolid nodules. These analyses may provide clues for the evolution of therapeutic strategies of lung cancer.

## Materials and methods

### Patient cohorts

**Fig. 1:**
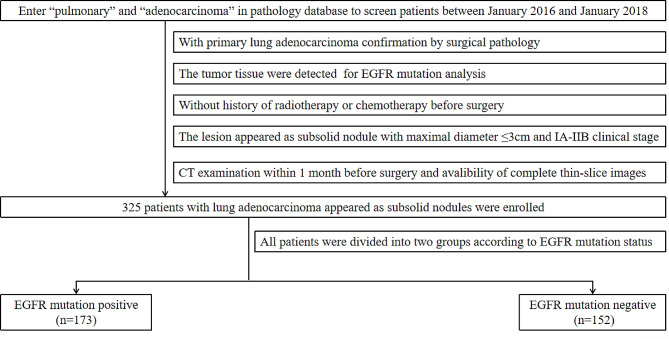
Patient inclusion flowchart shows the number of patients

This research obtained approvement form our Institutional Review Board. We retrospectively collected clinical staged IA to IIB patients who were pathologically confirmed as LUAD and had the lesion resected from January 2016 to January 2018 at the Chinese People’s Liberation Army General Hospital, Affiliated Beijing Shijitan Hospital of Capital Medical University and Affiliated Qingdao Hospital of Qingdao University. We selected patients with tumor presents as part-solid nodule and the maximal diameter were less than or equal to 3 cm. All patients had CT scan records within previous month before surgical operation. Patients with history of radiotherapy or chemotherapy before surgery were excluded because it might affect pathological changes and prognosis. Besides, those patients who did not undergo EGFR-mutation testing with resected surgical specimen were also excluded. The undetermined EGFR status would cause bias to the results. Finally, 325 patients were enrolled and analyzed in this study. Flowchart for patient selection were shown as Fig. 1. The clinical staging was based on the guidelines of the eighth edition of the TNM classification of the International Association for the Study of Lung Cancer [17].

### Radiological examination and assessment

The chest CT examination was taken in all patients. Radiological features were evaluated by two radiologists (C.W and T.J, both have over 15 years of working experience and were unaware of the mutation status of EGFR and histological subtype) on 1.0 ∼ 1.5 mm CT figures with lung window levels (WW, 1500 Hounsfield units (HU); WL, − 600HU) and mediastinal windows (WW, 350HU; WL, 40 HU). In case of disagreement among radiologists was resolved through discussion with another chest radiologist (J.W) who has more than 20 years’ experience until final consensus was reached. Registered CT characteristics included: location, tumor size (primary tumor’s mean diameter of the mightiest and tiniest in the axial plane), category of nodule (pGGN or mGGN), lobulation, spiculation, vascular changes, air-bronchogram, bubblelike lucency, pleural retraction.

### Pathological evaluation and EGFR mutation detection

The surgically resected tumor was fixed with formalin and made into paraffin-embedded tissue blocks. Routine slices (around 5 μm thick) without staining were prepared for DNA extraction. Then the extracted DNA was used for EGFR detection based on ARMS-PCR (Amplification Refractory Mutation System -Polymerase Chain Reactions). The accuracy and reliability of EGFR mutations were ensured by utilizing both upstream and downstream primers.

Hematoxylin and eosin-stained histopathological specimens were assessed by two well-experienced pulmonary pathologists (J.G. and X.X.). If there were disagreements, a joint session was convened to reach consensus. The histopathological diagnosis was determined as precursor glandular lesions or adenocarcinomas in the light of the WHO Classification of Lung Tumors in 2021 [18]. Tertiary lymphoid structures (TLS) were ectopic immune lymphoid aggregates, consisting of germinal centers comprised follicular dendritic cells, proliferative B cells, and a T cell region containing dendritic cells [19].. We examined all slides to evaluated TLS. In this study, the definition of TLS-positive (TLS+) cases has the following characteristics: lymphoid aggregates with visible germinal centers within the tumor area. Neither lymphoid aggregates nor lymphoid aggregates without germinal center in tumor region was defined as TLS-negative (TLS-) case. For TLS + cases, the number of tertiary lymphoid structures per unit tumor area was recorded.

### Patient follow-up

Patients regularly underwent physical examination, chest radiography, CT scans of brain, magnetic resonance imaging or blood tests, consisting of relevant tumor biomarkers after surgery. Any meaningful recurrence symptoms were recorded and further confirmed. Overall survival (OS) was defined as the length of time from the specific point, such as surgical resection, until the time of death. The recurrence-free survival (RFS) was considered to be the time interval from the day of surgery to initial recurrence. The cut-off date was January 2023 in this research. Finally, postoperative follow-up was available in 320 patients (98.5%) in this cohort. There is no death caused by lung cancer during the follow-up. Thus, we only analyzed RFS in this study.

### Statistical analysis

In this research, statistical data analysis was performed by SPSS Statistics (version 26.0, IBM Corporation, Armonk, NY). Continuous variables are described as means ± standard deviations with ranges. Categorical variables were tested using chi-quare or Fisher exact text, while normally distributed variables were tested using unpaired t-tests. Logistic regression model analysis was conducted to discriminate clinical pathological and radiological factors for EGFR mutation. The survival curve of RFS was estimated by the Kaplan-Meier methods. Cox proportional hazards analysis was performed to evaluate the prognostic impact of various factors (including clinical pathological, radiological features, tertiary lymphoid structures and EGFR mutation status), hazard ratios and 95% confidence intervals (CIs). P value < 0.05 means a statistically significant.

## Results

### Patient characteristics and EGFR mutations

A cohort of 325 patients with LUAD appearing as subsolid nodules were collected and analyzed. There were 209(64.3%) females and 116(35.6%) males, with average age 56.8 ± 9.8 years (between the ages of 22 and 80). There were 71(21.8%) patients who had smoking history. The mutation rate of EGFR at exon 21 was 37.2%, accounted for the most frequent mutation exon in 173 patients with EGFR mutation. Detailed clinical and EGFR mutation information are listed in Table 1.

**Table 1:** Characteristics of patient cohort

Characteristics	Number
Gender	
female	209(64.3%)
male	116(35.6%)
**Age**	
mean	56.8 ± 9.8
range	22–80
**Smoking history**	
positive	71(21.8%)
negative	254(78.2%)
**Clinical Stage**	
Stage I	315(96.9%)
Stage II	10(3.1%)
**EGFR mutation status**	
exon 18	7(2.2%)
exon 19	41(12.6%)
exon 20	2(0.6%)
exon 21	121(37.2%)
exon 19/21	2(0.6%)
wild-type	152(46.7%)

### Radiological and pathological features according to EGFR status

Table 2 displays the contrast in radiological and pathological characteristics among patients with EGFR mutation and those with wild type. Specifically, the EGFR mutations appeared of significant relevance with patients with lung mix ground glass nodules (*p*<0.01) and those with larger tumor diameters (*p*<0.01). Lobulation (*p* = 0.012), air-bronchograms (*p*<0.01), bubble-like lucency (*p*<0.01) and pleural retraction (*p* = 0.01) were more commonly indicated in tumors with EGFR mutations. The candidate variables identified by univariate analysis were analyzed using multivariate logistic regression in order to determine the independent factors associated with EGFR mutation (Table 3). The consequences of our study demonstrated that female(OR, 1.944; 95%CI, 1.136 ∼ 3.329; *p* = 0.015), mix ground glass nodule (OR, 2.071; 95%CI, 1.278 ∼ 3.357; *p* = 0.003) and bubble-like lucency (OR, 1.991; 95%CI, 1.268 ∼ 3.126; *p* = 0.003) were independent factors relevant to EGFR mutation.

**Table 2:** CT and pathological features according to EGFR mutation status

Characteristics	EGFR mutation (*n* = 173)	EGFR wild-type (*n* = 152)	*P*-values
Location			
right upper lobe	56(32.4%)	66(43.4%)	
right middle lobe	12(6.9%)	8(5.3%)	
right lower lobe	34(19.7%)	20(13.2%)	
left upper lobe	42(24.3%)	26(17.1%)	
left lower lobe	29(16.8%)	32(21.1%)	0.098
**Tumor size (cm)**	1.60 ± 0.50	1.24 ± 0.41	<0.01
**Category of nodule**			
pGGN	48(27.7%)	81(53.3%)	
mGGN	125(72.3%)	71(46.7%)	<0.01
**Lobulation**			
positive	90(52.0%)	59(38.8%)	
negative	83(48.0%)	93(61.2%)	0.012
**Spiculation**			
positive	49(28.3%)	38(25.0%)	
negative	124(71.7%)	114(75.0%)	0.497
**Vascular changes**			
positive	91(52.6%)	86(56.6%)	
negative	82(47.4%)	66(43.4%)	0.470
**Air-bronchogram**			
positive	81(46.8%)	54(35.5%)	
negative	92(53.2%)	98(64.5%)	0.034
**Bubblelike lucency**			
positive	98(56.6%)	63(41.4%)	
negative	75(43.4%)	89(58.6%)	<0.01
**Pleural retraction**			
positive	96(55.5%)	53(34.9%)	
negative	77(44.5%)	99(65.1%)	0.010
**Histopathologic subtype**			
precursor glandular lesions	5(2.9%)	6(3.9%)	
adenocarcinomas	168(97.1%)	146(96.1%)	0.597
**TLS**			
positive	76(43.9%)	92(68.4%)	
negative	97(56.1%)	60(31.6%)	<0.01

**Table 3:** Logistic regression model analysis for EGFR mutation in the cohort of 325 patients

Features	Status	Univariate analysis			Multivariate analysis		
		Odds ratio	95%CI	*P* value	Odds ratio	95%CI	*P* value
**Gender**	Female	1.995	1.342 ∼ 2.965	0.001	1.944	1.136 ∼ 3.329	0.015
**Age**	≥ 70	2.257	0.977 ∼ 5.213	0.057	NA		
**Smoking history**	negative	2.043	1.297 ∼ 3.218	0.002	1.607	0.869 ∼ 2.973	0.131
**Category of nodule**	mGGN	2.887	1.944 ∼ 4.287	< 0.001	2.071	1.278 ∼ 3.357	0.003
**Lobulation**	positive	1.709	1.159 ∼ 2.521	0.007	1.423	0.924 ∼ 2.191	0.110
**Spiculation**	positive	1.185	0.767 ∼ 1.832	0.444	NA		
**Vascular changes**	positive	0.852	0.580 ∼ 1.250	0.412	NA		
**Air-bronchogram**	positive	1.598	1.078 ∼ 2.368	0.020	0.969	0.615 ∼ 1.528	0.892
**Bubble-like lucency**	positive	1.846	1.254 ∼ 2.717	0.002	1.991	1.268 ∼ 3.126	0.003
**Pleural retraction**	positive	2.329	1.568 ∼ 3.458	< 0.001	1.477	0.931 ∼ 2.343	0.097

**Fig. 2:**
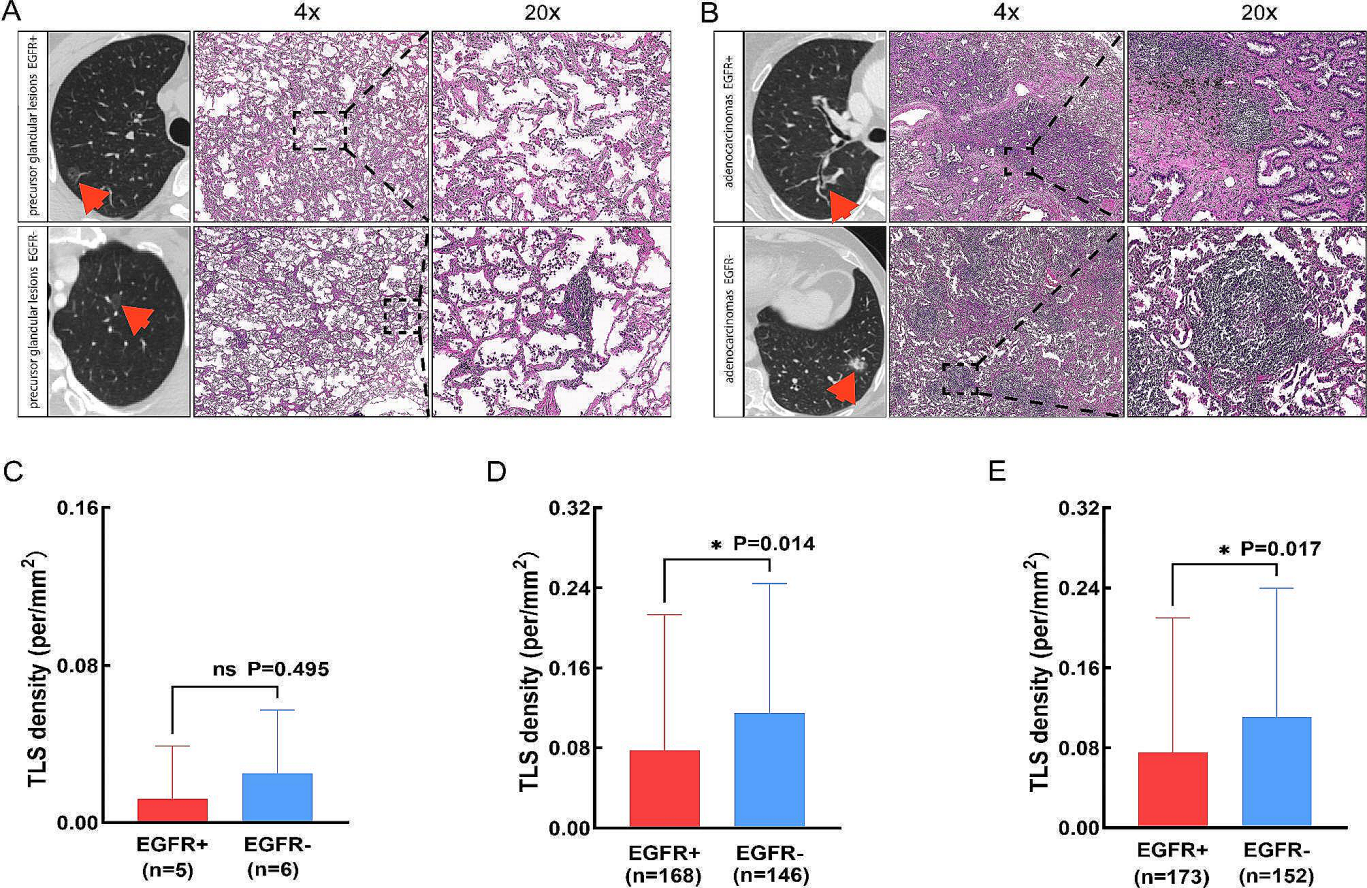
Assessment of tertiary lymphoid structures density according to pathological subtypes

From a pathological perspective, the proportion of TLS positive patients in the EGFR mutation group was lower than that in the wild-type group (*p*<0.01). Moreover, we analyzed the association between EGFR mutation and TLS density based on pathological subtypes and found that there were no significant differences in TLS density in patients with precursor glandular lesions (Fig. 2A and C). However, in patients with adenocarcinomas, the EGFR mutated patients tend to have lower TLS density(*p* = 0.014) than EGFR wild-type (Fig. 2B and D). As a whole, the TLS density was found to be lower among patients with EGFR mutation compared to those without (*p* = 0.017) (Fig. 2E).

### Survival outcomes according to EGFR status and TLS status

**Fig. 3:**
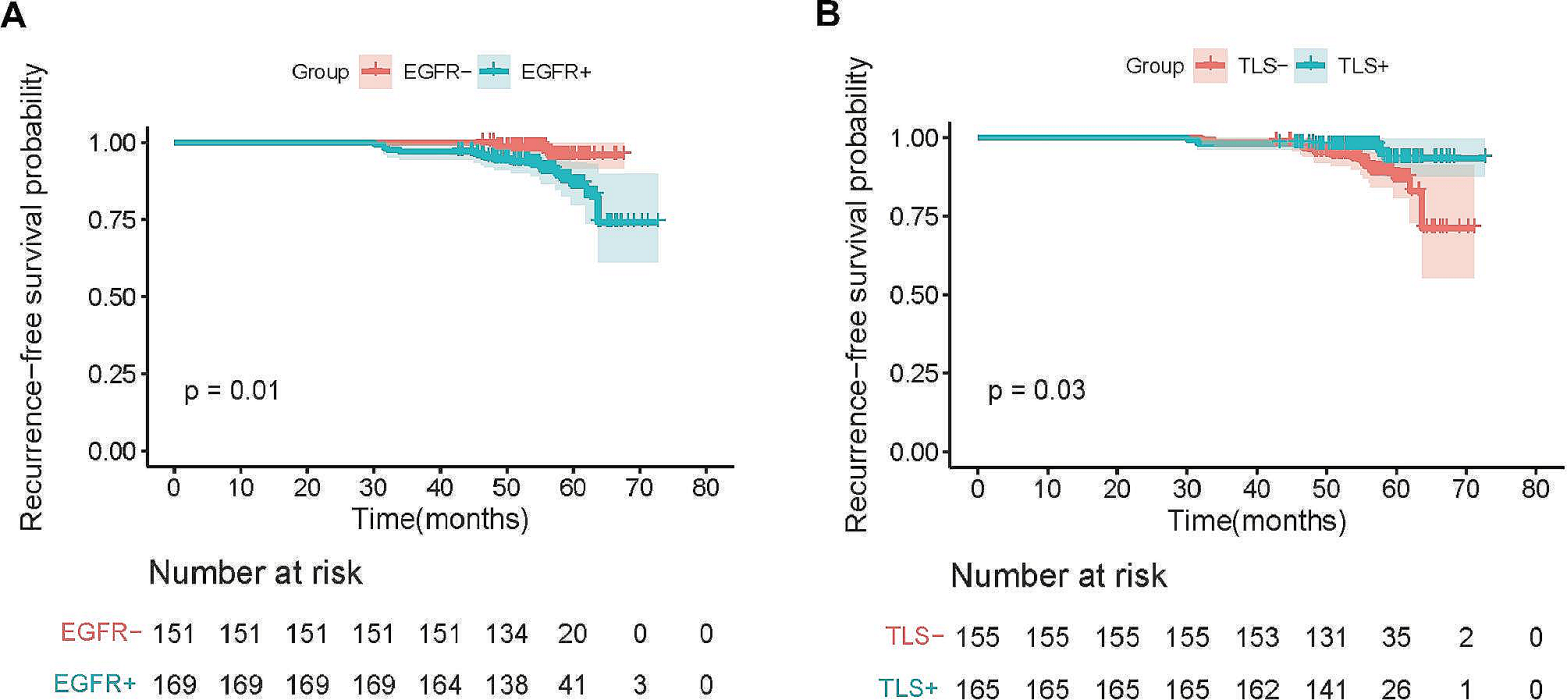
Kaplan-Meier analysis of RFS of 320 patients according to EGFR mutation status **(A)** and presence of tertiary lymphoid structures **(B)**

**Fig. 4:**
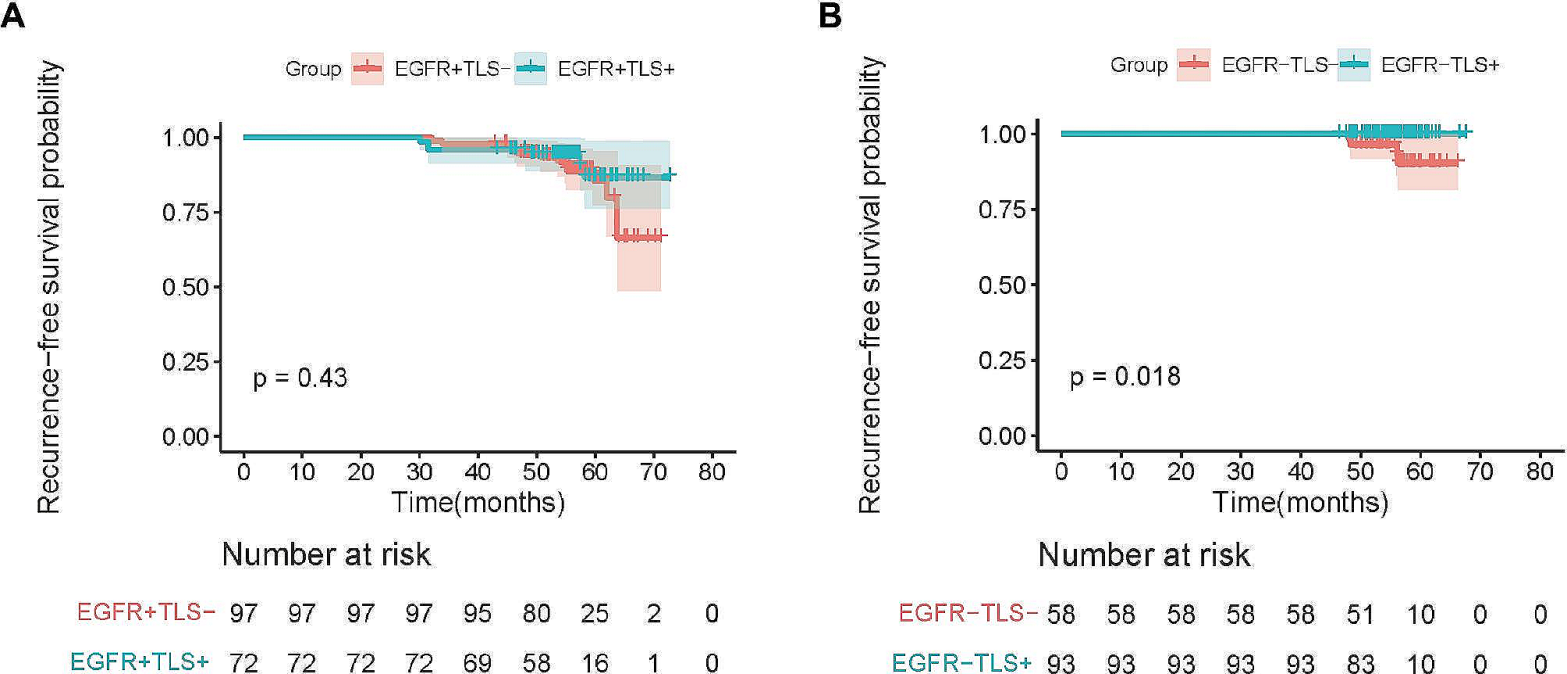
**(A)** Kaplan-Meier analysis of RFS of patients with presence of tertiary lymphoid structures (green line) and absence of tertiary lymphoid structures (red line) in the EGFR mutated group. **(B)** Kaplan-Meier analysis of RFS of patients with presence of tertiary lymphoid structures (green line) and absence of tertiary lymphoid structures (red line) in the EGFR wild-type group

The median follow-up duration in 320 cases is 55.6 ± 5.9 month. Median survival time was not reached, After the surgical procedure, adjuvant therapy was not administered to any of the patients. During the follow-up period, a total of 23 patients experienced recurrence, comprising 19 patients with EGFR mutation and 4 patients without. According to Kaplan-Meier analysis, the RFS was notably poorer in patients harboring EGFR mutations compared to those with wild-type EGFR(*p* = 0.01) (Fig. 3A). In additional, the RFS of patients with TLS + tended to be significantly better than that of patients with TLS-(*p* = 0.03) (Fig. 3B). We further analyzed the impact of TLS on RFS based on EGFR status. The Kaplan-Meier analyses confirmed that no statistically significant difference was observed in the TLS + and TLS- groups in patients with EGFR mutation (*p* = 0.43) (Fig. 4A). However, the RFS of patients with TLS + exhibited a statistically significant improvement compared to the TLS- group in patients without EGFR mutation (*p* = 0.018) (Fig. 4B). We performed the Cox proportional hazard analyses to ascertain prognostic factors influencing the RFS. As a result, univariate analyses of RFS revealed that EGFR mutation (HR, 3.674; 95%CI, 1.240 ∼ 10.883; *p* = 0.019) and TLS+ (HR, 0.380; 95%CI, 0.149 ∼ 0.965; *p* = 0.042) were significant prognostic factors for RFS. However, the multivariate analyses finally confirmed that EGFR mutation (HR, 3.205; 95%CI, 1.072 ∼ 9.581; *p* = 0.037) was independently remarkable prognostic factors for RFS (Table 4).

**Table 4:** Cox proportional hazards model analysis of RFS in 320 patients with follow-up

Features	Status	Univariate analysis			Multivariate analysis		
		Hazard ratio	95%CI	*P* value	Hazard ratio	95%CI	*P* value
**Gender**	Male	1.534	0.671 ∼ 3.506	0.310	NA		
**Age**	≥ 70	2.235	0.756 ∼ 6.603	0.146	NA		
**Tumor size**	1−2 cm	2.209	0.507–9.630	0.291	NA		
	2−3 cm	0.200	0.555–16.665	0.200	NA		
**Smoking history**	positive	2.367	0.944 ∼ 5.937	0.066	NA		
**Gene mutation**	positive	3.674	1.240 ∼ 10.883	0.019	3.205	1.072 ∼ 9.581	0.037
**Category of nodule**	mGGN	2.500	0.973 ∼ 6.426	0.057	NA		
**Lobulation**	positive	1.859	0.804 ∼ 4.300	0.147	NA		
**Spiculation**	positive	0.716	0.266 ∼ 1.929	0.508	NA		
**Vascular changes**	positive	1.006	0.442 ∼ 2.294	0.988	NA		
**Air-bronchogram**	positive	0.686	0.289 ∼ 1.629	0.393	NA		
**Bubble-like lucency**	positive	0.885	0.387 ∼ 2.019	0.771	NA		
**Pleural retraction**	positive	2.154	0.927 ∼ 5.006	0.074	NA		
**TLS**	positive	0.380	0.149 ∼ 0.965	0.042	0.451	0.176 ∼ 1.154	0.097

## Discussion

In recent years, EGFR driver mutations have been more frequently detected in LUAD, and targeted therapies against EGFR driver mutations have shown significant positive efficacy and tolerable toxicities [20, 21]. EGFR-TKIs are mainly used for advanced and metastatic NSCLC with EGFR mutations. Most of the past researches related to the influence of EGFR driver mutation only focused on the advanced stage of NSCLC [22–24]. However, as for early-stage lung cancer, the disparities in clinical features and survival outcomes due to the EGFR mutations had already generated [25, 26]. According to our research, we made an investigation on the impact of EGFR mutation on survival outcomes, tertiary lymphoid structures and radiological features in patients with stage I-II LUAD appearing as subsolid nodules. With the investigation progress on EGFR-TKI as neoadjuvant and adjuvant agents for EGFR-mutated NSCLC [27, 28], our research provided more evidence for necessity of treatment in earlier phase. Previous studies had controversy over the association among EGFR mutation and prognosis. A recent research conducted by the Japanese Joint Committee of Lung Cancer Registry reported that with EGFR mutation exhibited a significant improvement in DFS (HR, 0.894; 95%CI, 0.814–0.980; *p* = 0.017) and OS (HR, 0.729; 95%CI, 0.642–0.829; *p* < 0.001) in a cohort comprising 5780 patients who underwent surgical resection for early-stage NSCLC [29]. However, the Japan National Cancer Center Hospital East published a report that no differences were found in RFS and OS between lung cancer patients with GGO who had EGFR wild type and EGFR mutant status [30]. Similarly, another research also reported that the prognosis of NSCLC with pathological staging ranging from IB to III couldn’t been improved by EGFR mutation [31]. Notably, both the two studies indicated the trend that recurrence rate is higher in early-stage NSCLC with EGFR mutations. The results in our cohort demonstrated a significant association that patients with EGFR wild type showed a better RFS compared to patients with EGFR mutation under the condition of without postoperative therapy.

Tertiary lymphoid structure was considered to participate in activate antigen presentation and antitumor immune reaction, which was associated with favorable prognosis in NSCLC [32–36]. Zhou et al. have ever described the specific tumor microenvironment of LUAD with EGFR mutation from the perspective of cellular composition and function. They revealed that the EGFR wild-type group showed higher expression of CXCL13 (essential factor for the formation of TLS), as well as a higher density of TLSs [37]. The results in our cohort also corroborated the findings that EGFR mutation is negatively correlated with TLS formation and density. We found that both the proportion of patients with positive TLS and TLS density in EGFR mutant group was lower than EGFR wild group. Then we confirmed the patients with TLS + were inclined to have better RFS compared to patients with TLS-, with significant differences. Moreover, there was no difference in RFS between presence and absence of TLS in patient with EGFR mutation, but in early-stage lung cancer without EGFR mutations, TLS + patients had better RFS than TLS- patients. Although TLS is associated with favorable prognosis, the recurrence is more frequently observed in EGFR mutated lung cancer, the eventual offset of this consequence occurs, as for patients with EGFR mutation, the survival curves of TLS + and TLS- overlap. Finally, the multivariate analysis revealed the independent prognostic factor for RFS was EGFR mutation while TLS was not. We speculated that EGFR mutation suppressed the formation of TLS so that to weaken the impact of TLS on survival outcomes.

There were another several note-worthy findings in our cohorts. In early-stage LUAD, some clinical and radiological features did correlate with EGFR mutation. Consistent with former researches [38, 39], our study validated that female and non-smoker accounted for the vast majority of proportion in those EGFR mutated patients. In analysis of radiological features, the patients with EGFR mutation were significantly associated with bigger size of tumor, lobulation, air-bronchogram, bubblelike lucency and pleural retraction, and this observation aligned with formerly released studies [40–42]. Multivariate analysis demonstrated that gender, mGGN and bubblelike lucency were independent factors related to EGFR mutation. However, due to the tumor heterogeneity and small sample for EGFR detection, it is of great value to identify radiological features of patients with EGFR mutation and integrate the clinical variables into radiological characteristics to predict EGFR mutation before targeted therapy.

There are also limitations in our study. First, the long-term behavior of cancers could be influenced due to tumor heterogeneity and effect of surgical treatment. Second, the LUAD appeared as subsolid nodules usually show good prognosis after surgery. The follow-up in our cohort is relatively short, long-term survival outcomes are required to validate. Third, we hadn’t determined the relevance among prognosis and EGFR mutation subtypes in early-stage lung cancers.

In conclusion, early-stage LUAD appearing as subsolid nodule with EGFR mutation have specific clinical and radiological signatures. EGFR mutation was associated with worse survival outcomes after surgery and have negative correlation with TLS, which might weaken the positive impact of TLS on prognosis. Highly attention should be paid to the use of EGFR-TKI for further treatment as agents in early LUAD patients who carrying EGFR mutation.

## Data Availability

The dataset used and analyzed during the current study can be obtained from corresponding authors according to reasonable requirements.
